# Liraglutide prevents and reverses monocrotaline-induced pulmonary arterial hypertension by suppressing ET-1 and enhancing eNOS/sGC/PKG pathways

**DOI:** 10.1038/srep31788

**Published:** 2016-09-01

**Authors:** Mei-Yueh Lee, Kun-Bow Tsai, Jong-Hau Hsu, Shyi-Jang Shin, Jiunn-Ren Wu, Jwu-Lai Yeh

**Affiliations:** 1 Department of Pharmacology, College of Medicine, Kaohsiung Medical University, Kaohsiung, Taiwan; 2 Division of Endocrinology and Metabolism, Department of Internal Medicine, Kaohsiung Medical University Hospital, Kaohsiung, Taiwan; 3 Department of Internal Medicine, Kaohsiung Municipal Hsiaokang Hospital, Kaohsiung, Taiwan; 4 Department of Pathology, Kaohsiung Municipal Hsiaokang Hospital, Kaohsiung, Taiwan; 5 Department of Pediatrics, Kaohsiung Medical University Hospital, Kaohsiung, Taiwan; 6 Department of Pediatrics, College of Medicine, Kaohsiung Medical University, Kaohsiung, Taiwan; 7 Department of Marine Biotechnology and Resources, National Sun Yat-Sen University, Kaohsiung, Taiwan

## Abstract

Liraglutide, a glucagon-like peptide-1 receptor (GLP-1R) agonist, is widely used to treat diabetes. However, its effect on pulmonary arterial hypertension (PAH) is unknown. In this study, we investigated its effects on rats with monocrotaline (MCT)-induced PAH and mechanisms on rat pulmonary artery smooth muscle cells (PASMCs). Liraglutide was investigated for both prevention and treatment of MCT-induced PAH. The hemodynamic and body weight changes, right heart hypertrophy, lung morphology, immune-reactivity of endothelial nitric oxide synthase (eNOS), endothelin-1 and cyclic guanosine monophosphate (cGMP) levels, protein expressions of eNOS, soluble guanylyl cyclase (sGCα), protein kinase G (PKG) and Rho kinase (ROCK) II pathway were measured in both *in vivo* and *in vitro*. Cell migration and cell cycle were also determined. Liraglutide both prevented and reversed MCT-induced PAH, right ventricle hypertrophy and pulmonary vascular wall remodeling. Protein expression of ROCK II was increased while eNOS, sGC and PKG were decreased. Pretreatment with liraglutide inhibited platelet-derived growth factor (PDGF)-BB stimulated PASMCs migration, which were associated with cell-cycle arrest at G_0_/G_1_ phase. Liraglutide may have both preventive and therapeutic effects on MCT-induced PAH, through the eNOS/sGC/PKG and Rho kinase pathways. Thus, liraglutide may have a therapeutic role in pulmonary vascular remodelling.

Recent studies suggest that diabetes is a risk factor for pulmonary arterial hypertension (PAH)^1^. Endothelial dysfunction, classically characterized by a reduced capacity of endothelial cells to induce vasodilatation via the release of nitric oxide (NO), is an early and independent predictor of a poor prognosis of PAH^2^,^3^. The signaling pathway of NO, cGMP, and cGMP-dependent protein kinases has been shown to be down regulated under diabetic conditions and to contribute to the development of diabetic vascular complications^4^.

PAH is a disease often considered to be driven by vasoconstriction. It is well established that the endothelin, NO and prostacyclin pathways play important roles in the development of PAH. Endothelin-1 (ET-1) is a key mediator of PAH, released from the endothelium, driving pathological changes in the lung that lead to pulmonary vascular remodeling^5^. PAH is associated with impaired production of the endothelium-derived vasodilator, NO^6^. eNOS is a nitric oxide synthase that generates NO in blood vessels and is involved with regulating vascular tone by inhibiting smooth muscle contraction and platelet aggregation. In healthy individuals, NO acts on smooth muscle cells to induce vasodilation and inhibit proliferation by increasing production of the secondary messenger cGMP via activation of soluble guanylate cyclase (sGC)^7^, which is the only known receptor for NO^8^. cGMP-dependent protein kinase or protein kinase G (PKG) is implicated in the regulation of smooth muscle relaxation, platelet function, sperm metabolism, cell division, and nucleic acid synthesis^9^. Rho-associated protein kinase (ROCK) have been reported to be the first effector of Rho and to induce the formation of stress fibers and focal adhesions by phosphorylating myosin light chains, actin binds to myosin II and, thus, the contractility increases^10^. Indeed, the therapeutic options target one of these mechanistic pathways may be of benefit in PAH.

Liraglutide, an analogue of GLP-1, which is an incretin and a peptide hormone that stimulates insulin and inhibits glucagon secretion in a glucose-dependent manner^11^. GLP-1 targets multiple organs to improve glucose control in patients with type 2 diabetes mellitus^12^, and has been found to exert favorable actions on cardiovascular function in preclinical and clinical studies. GLP-1 increases myocardial glucose uptake during aerobic perfusion, independent of insulin-stimulated Akt phosphorylation and glucose transporter 4 (GLUT4) translocation, in association with increased p38 mitogen-activated protein kinase (MAPK) activity, enhanced NO production, and increased GLUT1 protein levels at the sarcolemmal membrane^12^.

Current therapies for chronic pulmonary hypertension are designed to reduce pulmonary arterial resistance by inducing vasodilation (e.g., NO inhalation, stimulation of cGMP production by phosphodiesterase inhibitors, endothelin receptor antagonists, and prostacyclin analogs). However, these therapeutic approaches mainly provide symptomatic relief, and novel treatments are required to prevent the progression of pulmonary hypertension by interfering with the pathomechanisms of the disease at multiple levels to exert antimitogenic effects on the proliferation of pulmonary arterial smooth muscle cells (PASMCs). The aims of this study were to investigate the effects of liraglutide on monocrotaline (MCT) induced PAH in rats and its influence on the protein expressions of eNOS, sGC, PKG and Rho kinase of lung tissue and PASMCs.

## Results

### Effects of liraglutide on body weight, mean arterial blood pressure, mean heart rate, mPAP and glucose level

Body weight was measured before and after a single dose of MCT (60 mg·kg^−1^, i.p.) in the prevention (n = 24) and treatment (n = 20) groups after 21 days and 42 days, respectively. After 21 days, there were significant reductions in body weight in the Pv saline group (n = 8), Pv 75 (n = 8) and Pv 200 (n = 8) groups compared with the normal controls (n = 8) (400.38 ± 14.10 g, 342.00 ± 13.60 g, 329.75 ± 14.58 g and 325.00 ± 9.62 g, respectively, all p < 0.05). After 42 days, compared with the Tx saline group (n = 5), there was no significant change in body weight in the Tx 75 (n = 7) or Tx 200 (n = 8) groups. Compared with the normal control group and Pv saline, Pv 75 and Pv 200 groups had significantly decreased mean arterial blood pressure (122.21 ± 3.52 mmHg, 117.34 ± 1.91 mmHg, 110.07 ± 1.81 mmHg and 104.51 ± 1.59 mmHg, respectively, p < 0.05). Compared with the Tx saline group, the Tx 75 and Tx 200 groups had significant decreases in mean arterial blood pressure (120.62 ± 2.90 mmHg, 108.50 ± 3.06 mmHg and 110.11 ± 1.39 mmHg, respectively, p < 0.05). There were no significant differences in mean heart rate or glucose level in the prevention or treatment groups. However, compared with the normal control group, there was a significant elevation of mPAP in the Pv saline group (15.51 ± 1.58 mmHg vs. 27.80 ± 2.79 mmHg, p < 0.05), and this elevation in mPAP was significantly reduced in the Pv 75 and Pv 200 groups (18.47 ± 2.29 mmHg and 16.22 ± 1.25 mmHg, p < 0.05). This effect was also observed in the treatment groups, and the elevation of mPAP seen in the Tx saline group was significantly reduced in the Tx 75 and Tx 200 groups (37.64 ± 5.25 mmHg vs. 25.52 ± 2.30 mmHg and 22.24 ± 2.22 mmHg, p < 0.05). After fully adjusted of mPAP with body weight, mABP, heart rate and glucose level in prevention group, there was a significantly reduced of mPAP in Pv 200 groups compared with Pv saline group (p < 0.05, 95% confidence interval, −33.6~−6.36), but not among treatment group.

### Effects of liraglutide in PAH-associated RVH, pulmonary wall thickening and medial thickness

**Figure 1:**
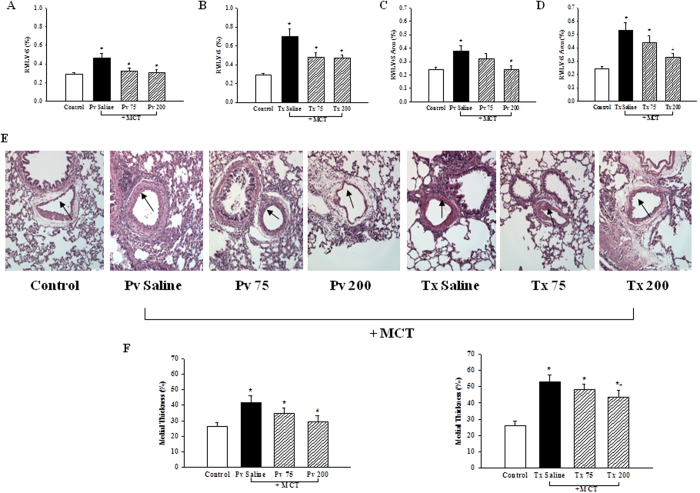
Effects of GLP-1receptor agonist liraglutide on MCT-induced right ventricular hypertrophy (RVH) (**A**–**D**), (**E**) pulmonary vascular morphology, (**F**) medial thickness. Both liraglutide 75 and 200 μg·kg^−1^ (**A**,**C**) prevention (**B**,**D**) treatment groups improved MCT-induced RVH represented by weight and area ratio. Liraglutide and saline were intraperitoneally administered twice daily for 3 weeks. Long-term treatment with liraglutide after the injection of MCT reduced the wall thickness of pulmonary artery. Pulmonary arteries were stained with hematoxyline and eosin (**E**). Bars represent the mean ± SEM of n = 5 per group. *p < 0.05 versus controls; ^#^p < 0.05 versus saline prevention group; ^+^p < 0.05 versus saline treatment group (**F**). MCT = monocrotaline; LV = left ventricle; Pv saline = saline prevention group; Pv 75 = liraglutide 75 μg·kg^−1^ prevention group; Pv 200 = liraglutide 200 μg·kg^−1^ prevention group; Tx saline = saline treatment group; Tx 75 = liraglutide 75 μg·kg^−1^ treatment group; Tx 200 = liraglutide 200 μg·kg^−1^ treatment group; scale bar = 100 μm. RV = right ventricle; S = septum.

In the Pv saline group, the degree of RVH was significantly higher than in the normal control group due to the effect of MCT-induced PAH (0.46 ± 0.07% vs. 0.29 ± 0.02%, p < 0.05) (Fig. 1A). However, this effect was abated in the Pv 75 (0.32 ± 0.06%) and Pv 200 (0.24 ± 0.03%) groups (p < 0.05, Fig. 1A). This deterioration was also significantly prevented in the Tx 75 and Tx 200 groups compared with the Tx saline group (0.70 ± 0.14%, 0.48 ± 0.05% and 0.47 ± 0.03%, P < 0.05) (Fig. 1B). On the other hand, the relative weight ratio was found to be significantly increased in the Pv saline group compared with the normal control group (0.24 ± 0.02 vs. 0.38 ± 0.06, p < 0.05) (Fig. 1C). The RVH induced by MCT was effectively improved in the Pv 200 group (0.24 ± 0.03, p < 0.05). Similarly, the RVH was effectively improved in the Tx 200 group compared with the Tx saline group (0.53 ± 0.13 vs. 0.33 ± 0.03, p < 0.05) (Fig. 1D). As shown in Fig. 1E, there was obvious hyperplasia and hypertrophy in the pulmonary arteries of the Pv saline group compared with the normal control group after administration with MCT. However, the proliferation of pulmonary arteries in the Pv 75 and Pv 200 groups were improved compared with the Pv saline group. This finding was also observed in the Tx 75 and Tx 200 groups compared with Tx saline group. As shown in Fig. 1F, the percentage of pulmonary artery medial thickness in the normal control group was 26.11 ± 2.50%, which was significantly increased to 41.65 ± 6.67% in the Pv saline group (p < 0.05). However, the MCT-induced vascular hypertrophy was prevented in the Pv 200 group (29.46 ± 3.80%, p < 0.05). Compared with the Tx saline group, MCT-induced vascular hypertrophy was reduced in the Tx 200 group (52.92 ± 4.37% vs. 43.42 ± 6.32%, p < 0.05).

### Effect of liraglutide on the plasma concentration of ET-1

**Figure 2:**
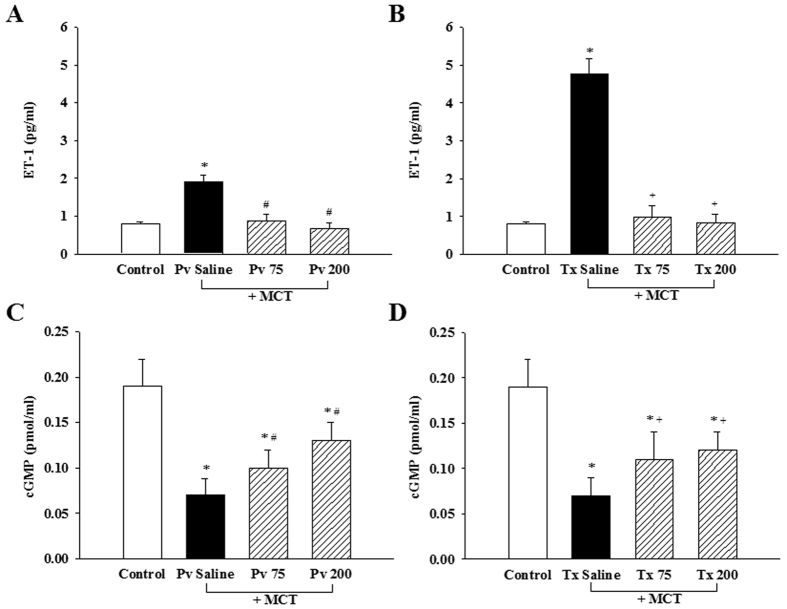
Effects of liraglutide on ET-1 concentration changes in rat plasma obtained by cardiac puncture and cGMP concentration changes in rat lung tissue. Plasma ET-1 and rat lung tissue cGMP concentrations were decreased after liragltuide treatments compared to saline. Bars represent the mean ± SEM of n = 5 per group. *p < 0.05 versus controls; ^#^p < 0.05 versus saline prevention group; ^+^p < 0.05 versus saline treatment group. MCT = monocrotaline; cGMP = cyclicguanosine monophosphate; ET-1 = endothelin-1; Pv saline = saline prevention group; Pv 75 = liraglutide 75 μg·kg^−1^ prevention group; Pv 200 = liraglutide 200 μg·kg^−1^ prevention group; Tx saline = saline treatment group; Tx 75 = liraglutide 75 μg·kg^−1^ treatment group; Tx 200 = liraglutide 200 μg·kg^−1^ treatment group.

**Figure 3:**
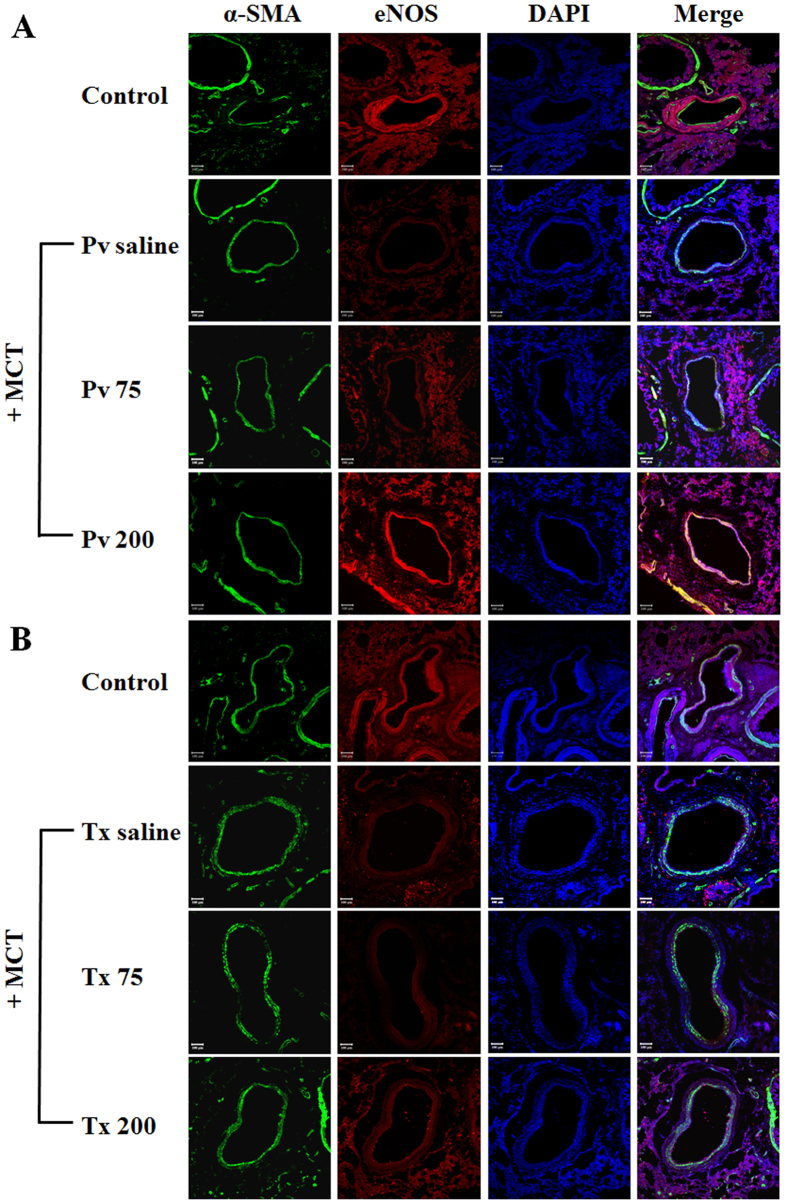
Confocal microscopy of eNOS in the control group and in rats treated (**A**) preventively after 21 days and (**B**) post-treated after 42 days with saline, liraglutide 75 and 200 μg·kg^−1^. Following a single intraperitoneal injection of monocrotaline (60 μg·kg^−1^) on day 1 and day 21, saline, liraglutide 75 μg·kg^−1^ and 200 μg·kg^−1^ were administered intraperitoneally twice daily for 3 weeks. MCT = monocrotaline; DAPI = 4′,6-diamidino-2-phenylindole; eNOS = endothelial nitric oxide synthase; αSMA = smooth muscle artery; Pv saline = saline prevention group; Pv 75 = liraglutide 75 μg·kg^−1^ prevention group; Pv 200 = liraglutide 200 μg·kg^−1^ prevention group; Tx saline = saline treatment group; Tx 75 = liraglutide 75 μg·kg^−1^ treatment group; Tx 200 = liraglutide 200 μg·kg^−1^ treatment group; scale bar = 100 μm.

ET-1 plays a role in the inhibition of eNOS and stimulates Rho kinase activation. Compared with the normal control group, the plasma concentration of ET-1 was significantly increased in the Pv saline group (0.80 ± 0.05 ng·mL^−1 ^vs. 1.91 ± 0.18 ng·mL^−1^, p < 0.05) (Fig. 2A), and this increase in ET-1 was significantly reduced in the Pv 75 and Pv 200 groups (0.88 ± 0.40 ng·mL^−1^ and 0.68 ± 0.15 ng·mL^−1^, p < 0.05) (Fig. 3A). Compared with the Tx saline group, the level of ET-1 was also clearly reduced in the Tx 75 and Tx 200 groups (4.76 ± 0.90 ng·mL^−1^, 0.99 ± 0.30 ng·mL^−1^ and 0.82 ± 0.23 ng·mL^−1^, p < 0.05) (Fig. 2B), which shows that liraglutide was effective in preventing and treating the increase in plasma ET-1 caused by MCT.

### Effect of liraglutide on cGMP level

cGMP was also inhibited in the lungs of the rats by MCT. Compared with the normal control group, the cGMP level in rat lung tissues was significantly reduced in the Pv saline group (0.19 ± 0.14 pmol·mL^−1^ and 0.07 ± 0.018 pmol·mL^−1^, p < 0.05) (Fig. 2C). This effect was reversed in the Pv 75 and Pv 200 groups (0.10 ± 0.03 pmol·mL^−1^ and 0.13 ± 0.03 pmol·mL^−1^, p < 0.05) (Fig. 2C). This was also clearly observed in the treatment group. Compared with the Tx saline group, the levels were reversed in the Tx 75 and Tx 200 groups (0.07 ± 0.02 pmol·mL^−1^, 0.11 ± 0.04 pmol·mL^−1^ and 0.12 ± 0.02 pmol·mL^−1^, p < 0.05) (Fig. 2D).

### Effect of liraglutide on eNOS expressions

Immunocytochemistry of eNOS expression in the pulmonary arteries of the rats showed decreased eNOS expressions with MCT treatment in the Pv and Tx saline groups compared with the normal control group. In addition, the eNOS expression in rat pulmonary artery endothelium was reversed to nearly the level of the normal control group in the Pv and Tx 200 groups (Fig. 3A,B).

### Effects of liraglutide on the protein expressions of eNOS, sGC, PKG and ROCK II *in vivo*

**Figure 4:**
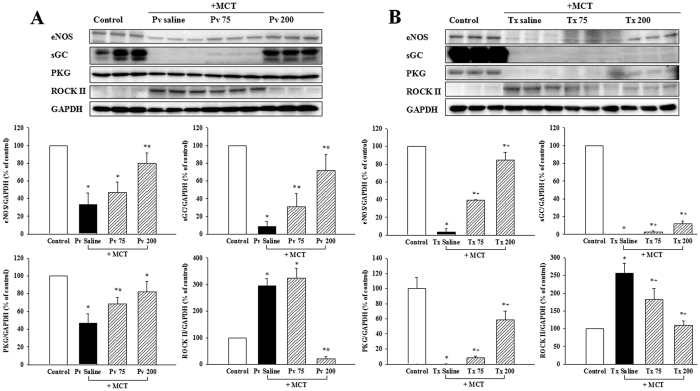
Effects of liraglutide on MCT-induced expression of, eNOS, sGC, PKG, and ROCK II in lung tissues of (**A**) prevention and (**B**) treatment group shown in cropped blots. Each value represents the mean ± SEM of n = 6. *p < 0.05 versus controls; ^#^p < 0.05 versus saline prevention group; ^+^p < 0.05 versus saline treatment group. MCT = monocrotaline; eNOS = endothelial nitric oxide synthase; PKG = protein kinase G; ROCK II = Rho kinase II; sGC = soluble guanylate cyclase; Pv saline = saline prevention group; Pv 75 = liraglutide 75 μg·kg^−1^ prevention group; Pv 200 = liraglutide 200 μg·kg^−1^ prevention group; Tx saline = saline treatment group; Tx 75 = liraglutide 75 μg·kg^−1^ treatment group; Tx 200 = liraglutide 200 μg·kg^−1^ treatment group.

The eNOS expression in the rat lung tissues were significantly reduced in the Pv and Tx saline groups compared with the normal control group, and this effect was reversed in the Pv 200, Tx 75 and Tx 200 groups (Fig. 4A,B). In addition, the protein expressions of sGC and PKG were also inhibited by MCT and significantly reduced in rat lung tissues of the Pv and Tx saline groups compared with the normal control group. This effect was reversed in the Pv 200, Tx 75 and Tx 200 groups (Fig. 4A,B). On the other hand, the expression ROCK II was significantly increased in rat lung tissues in the Pv and Tx saline groups, while ROCK II expression levels were reduced in the Tx 75, Tx 200, and Pv 200 groups (Fig. 4A,B; Supplementary information).

### Effect of liragltutide on PDGF-BB-induced PASMC migration

**Figure 5:**
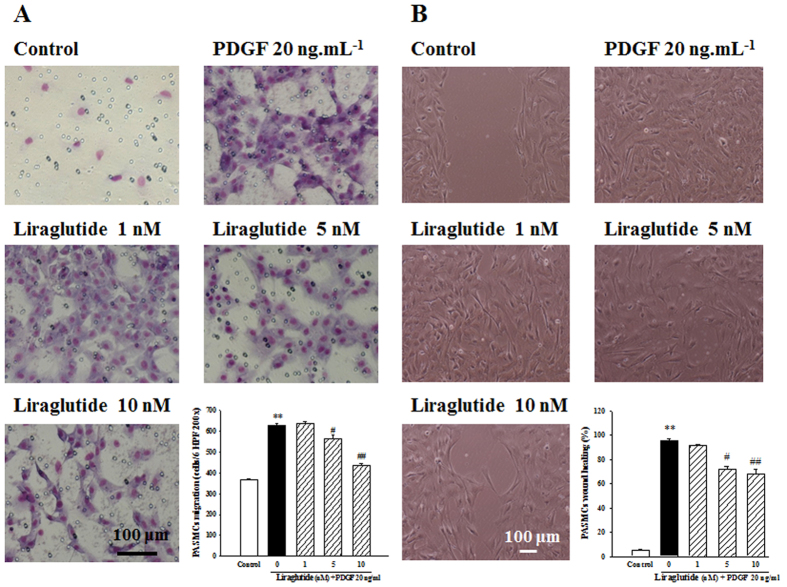
Effects of liraglutide on cell migration in Boyden chamber and wounding assays. (**A**) PDGF-BB induced migration of rat PASMCs into the upper chamber from the lower chamber at 24 h, Liraglutide inhibited PDGF-BB-induced migration dose-dependently. The bar graph shows migration activities assayed in quadruplicate in three independent experiments for the number of cells observed in six high-power fields. (**B**) Wound closure was photographed (top) and measured under microscopy (HPF × 100) (bottom) by wound-healing assay. Values represent the mean ± SEM of n = 3–4. Control: PASMCs were placed in medium with 1% FBS. ^*^p < 0.05 versus control group; ^#^p < 0.05 versus cells exposed to PDGF-BB alone.

To elucidate the anti-migratory effects of liraglutide, we performed two types of migration assay. We first examined whether liraglutide pretreatment (1–100 ng·mL^−1^, 1 h) affects PDGF-BB (20 ng·mL^−1^)-induced PASMC migration by using Boyden chamber assay. As shown in Fig. 5A, PDGF-BB-induced cell migration was significantly inhibited by liraglutide in a concentration-dependent manner. Next, we assessed the inhibitory effect of liraglutide on cell migration in the artificial scratch wound of PDGF-BB-stimulated PASMC. As shown in Fig. 5B, liraglutide significantly reduced cell numbers in the scratch wound area for PDGF-BB-induced PASMC.

### Effect of liralgutide on PDGF-BB-stimulated cell arrest in the G_0_/G_1_-phase

**Figure 6:**
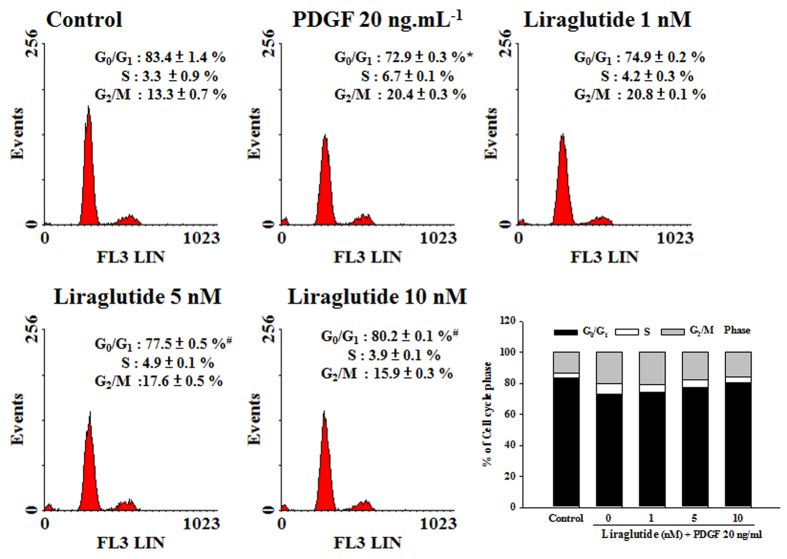
Effects of liraglutide on cell cycle progression affected by PDGF-BB. Liraglutide increased the percentage of PASMCs in the G_0_/G_1_-phase dose-dependently. Quiescent cells were pretreated with liraglutide (1–10 nM) for 1 h followed by the addition of PDGF-BB and incubated for 48 h. Values represent the mean ± SEM of n = 3–4. *p < 0.05 versus control group; ^#^p < 0.05 versus cells exposed to PDGF-BB alone.

The effects of liraglutide on cell cycle progression were influenced by PDGF-BB (Fig. 6). In this study, 83.4 ± 1.4% of the cells arrested in the G_0_/G_1_-phase of the cell cycle were rendered quiescent by serum starvation. After PDGF-BB treatment for 48 h, the percentage of cells in the G_0_/G_1_-phase significantly decreased to 72.9 ± 0.3% (p < 0.01). Liraglutide also inhibited the PDGF-BB-induced decrease in the G_0_/G_1_-phase in a dose-dependent manner (p < 0.05). Thus, liraglutide might have the potential of arresting the PDGF-BB-stimulated PASMCs in the G_0_/G_1_-phase.

### Effects of liraglutide on the protein expressions of eNOS, sGC, PKG and ROCK II *in vitro*

**Figure 7:**
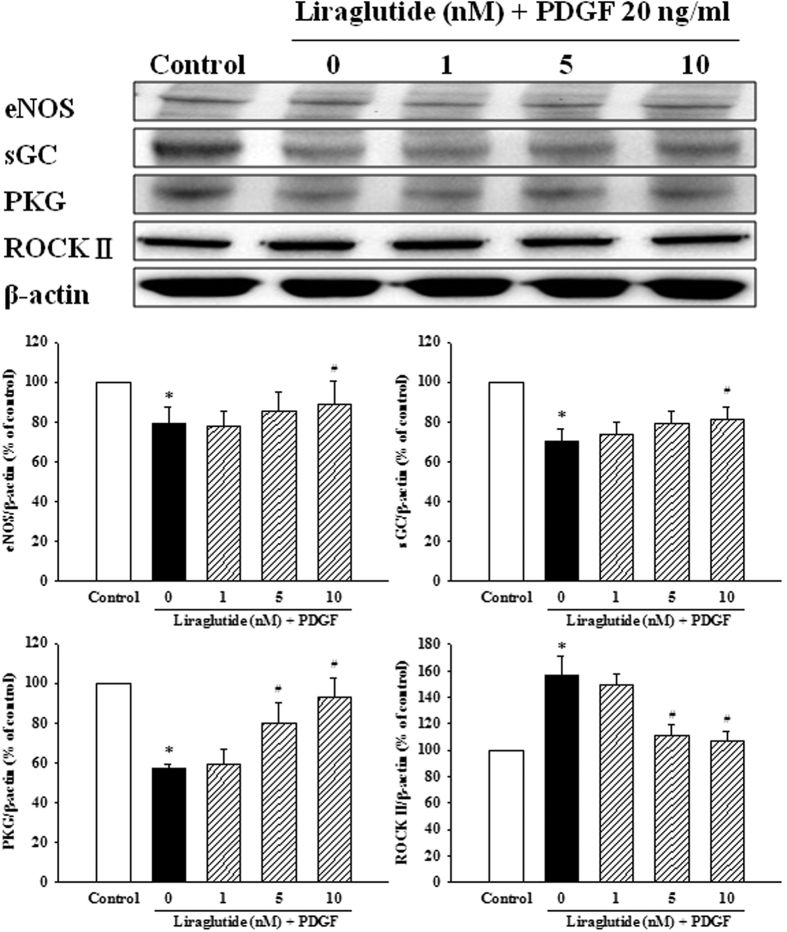
Effects of liraglutide on PDGF-BB-induced expression of, eNOS, sGC, PKG, and ROCK II in rat PASMCs shown in cropped blots. Values represent the mean ± SEM of n = 3–4. ^*^p < 0.05 versus control group; ^#^p < 0.05 versus cells exposed to PDGF-BB alone. eNOS = endothelial nitric oxide synthase; PASMC = pulmonary artery smooth muscle cell; PDGF-BB = platelet derived growth factor; PKG = protein kinase G; ROCK II = Rho kinase II; sGC = soluble guanylate cyclase.

In PASMCs, the eNOS and sGC expression were significantly reduced in the PDGF-BB alone compared with the normal control group, and this effect was reversed in the liraglutide 10 nM (Fig. 7). In addition, the protein expression of PKG was also inhibited by PDGF-BB alone compared with the normal control group. This effect was reversed in the liraglutide 5 and 10 nM. On the other hand, the expression ROCK II was significantly increased PDGF-BB alone, while ROCK II expression level was reduced in the liraglutide 5 and 10 nM (Fig. 7; Supplementary information).

## Discussion

**Figure 8:**
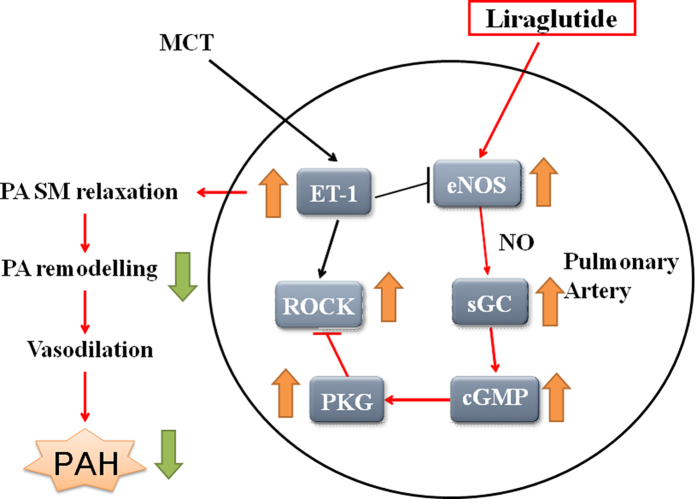
Proposed mechanism of action of liraglutide on rat lung tissues. Liraglutide activates the NO/sGC/cGMP pathways and subsequently increases the intracellular levels of cyclic GMP. Increased cGMP then activate the protein kinase PKG cascades, and thus reduces the Rho kinase expression, leading to attenuation of myocyte proliferation and hyperplasia. cGMP = cyclic guanosine monophosphate: ET-1 = endothelin-1: eNOS = endothelial nitric oxide synthase; MCT = monocrotaline: NO = nitric oxide: PAH = pulmonary artery hypertension; PKG = protein kinase G; ROCK = Rho kinase; sGC = soluble guanylate cyclase: SM; smooth muscle.

In this study, liraglutide showed both preventive and therapeutic effects on MCT-induced PAH in rats. The mechanisms by which liraglutide inhibit PAH may be due to activation of eNOS and sGC in PA endothelium, and inhibition of ROCK II in PASMCs by liraglutide may protect against MCT-induced chronic PAH over long-term administration. The inhibition of endothelial ROCK by liraglutide can initiate eNOS expression, and PKG can be increased by liraglutide-induced enhancement of eNOS, activation of sGC and inhibition of ROCK (Fig. 8).

Pulmonary hypertension refers to the hemodynamic state in which the pressure measured in the pulmonary artery is elevated by a mPAP of greater than 25 mmHg in the setting of normal or reduced cardiac output and a normal pulmonary capillary wedge pressure^13^. The treatment of pulmonary hypertension includes lifestyle modifications, conventional treatments such as diuretics and warfarin anticoagulation, and disease-specific treatments such as calcium channel blockers, prostacyclins such as intravenous epoprostenol, subcutaneous treprostinil, aerosolized iloprost and oral beraprost, and endothelin receptor antagonists such as bosentan which is an orally active, nonselective endothelin receptor antagonist, and sitaxsentan and ambrisentan, ET_A_ selective antagonists, and the known potent and highly specific phosphodiesterase type 5 inhibitor, sildenafil^6^. The goals of treatment are to alleviate symptoms, improvement the quality of life and survival. However, no known agent can prevent or treat pulmonary hypertension.

Studies have shown that the toxic alkaloid MCT in rats resulted in elevated right ventricular systolic pressure, right ventricular hypertrophy, increased pulmonary vessel wall thickness, interstitial fibrosis and PAH. These changes were associated with increases in the mRNA levels of renin, angiotensin converting enzyme (ACE), angiotensinogen, angiotensin II receptor type 1 (AT1) receptors, and proinflammatory cytokines, which might have a role in the development of PAH^14^,^15^,^16^ some studies have also shown that MCT causes increased plasma ET-1 concentrations in rats^17^,^18^. Other studies have shown that an increase in ET-1 concentration can inhibit NO production decrease the expression of eNOS^19^, and then activate Rho kinase^20^. In this study, we used MCT to induce PAH to determine whether liraglutide can inhibit PAH induced by MCT. Our results showed that after 21 days of single dose injections of MCT, pulmonary artery pressure was significantly higher in the absence of liraglutide treatment in the saline groups and after 21 days of intraperitoneal injections of liraglutide twice a day significantly improved PAH, showing that liraglutide had prevention and treatment effects on MCT-induced PAH. In addition, based on our results of plasma ET-1 concentrations, compared with the normal control group, the saline group had an increase in plasma ET-1 concentration, which is consistent with the previous studies. Interestingly, the administration of liraglutide significantly reduced the increased concentration of ET-1. In the analysis of lung and heart morphology, liraglutide was found to improve the medial wall thickening of the rat pulmonary arteries, right ventricle hypertrophy and, RV/LV + septum weight and area ratio induced by MCT, which is consistent with other studies^21^.

GLP-1 analogues are a new class of anti-diabetic drugs that is glucose dependent, the higher the plasma glucose level, the greater the effect of GLP-1 on insulin secretion with the greatest effect in hyperglycemic conditions, and little or no effect when the blood glucose concentration is less than 3.6 mmol·L^−1^ 22. Hence they do not themselves cause hypoglycemia. In the current study, no significant changes in glucose level were noted in the liraglutide-treated rats compared with the control and saline treated rats. GLP-1 analogues are associated with a small increase in heart rate and modest reductions in body weight and blood pressure^23^. In the current study, there was a reduction in body weight and blood pressure in the liraglutide-treated rats compared with the control and saline treated rats. However, no significant change was noted in heart rate.

As shown in Western blot analysis of lung tissue and immunocytochemistry analysis of PA eNOS, MCT reduced the eNOS expression, however liraglutide reversed this reduction. This may be due to liraglutide decreasing the plasma and lung tissue concentrations of ET-1, which is also consistent with other studies^24^. Other proteins such as sGC and PKG were also significantly suppressed by MCT and improved by liraglutide both in the prevention and treatment groups. According to previous studies, the Rho kinase protein is overexpressed by MCT^25^. In the current study, liraglutide showed prevention and treatment effects on the overexpression of ROCK II. Rho kinase in the Rho kinase-mediated pathway is associated with the formation of PAH, and Rho kinase is known to be activated by various activating factors of the blood vessels such as angiotensin II, ET-1, and serotonin, resulting in vascular smooth muscle contraction and proliferation, and ultimately PAH. Liraglutide suppressed the expression of ROCK II which may have been due to a decrease in plasma and lung tissue ET-1 concentrations and expression, resulting in a reduction of Rho kinase activation. The inhibitory effect of eNOS was not sufficient to completely reduce the expression of ET-1, and therefore a large amount of NO was produced which activated the sGC/PKG pathway to further enhance the role in the prevention and treatment of PAH by liraglutide.

In the current study, the action of liraglutide in MCT-induced PAH was probably via the NO/sGC/cGMP pathway to induce vascular relaxation. The increase in the release of NO activated sGC causing further increased synthesis of cGMP^26^, which is very important in regulating vascular tension, to avoid vascular remodeling and reduce pulmonary hypertension^27^. In the current study, liraglutide was found to prevent and treat symptoms of worsening PAH. The selectivity of the pulmonary vascular system in the treatment of PAH is the advantage of liraglutide. In our experiments, liraglutide was found to enhance eNOS expression, increase cGMP levels, and inhibit Rho kinase. In addition, liraglutide also had an effect on preventing ET-1 over-expression. Study shows that activation of pulmonary ACE2 enzyme by a small synthetic molecule XNT (1-[(2-dimethylamino)ethylamino]-4-(hydroxymethyl)-7-[[(4-methylphenyl)sulfonyl]oxy]-9H-xanthene-9-one) prevents pulmonary hypertension and vascular remodeling, and suggests that pulmonary ACE2 may be a novel target for the successful control of pulmonary hypertension^16^. In addition of some studies showed activation of GLP-1 receptor by liraglutide increases ACE2 expression and preservation of GLP-1 is associated with a reduction of angiotensin II-induced cardiac fibrosis, and reversing right ventricular hypertrophy in type 1 diabetes rats^28^,^29^. We conclude that liraglutide has a great potential as an anti-pulmonary hypertension drug.

Future studies are needed to determine other effects of liraglutide against PAH such as the mechanism of regulating serotonin, and the effects of liraglutide against PAH in diabetic rats. In addition, further studies are required to clarify its association with cellular, molecular, and genetic mechanisms. In humans, further studies should explore the effects of liraglutide on PAH markers such as brain natriuretic peptide and high-sensitive C-reactive protein, to further confirm that liraglutide may be used in the clinical treatment of PAH.

## Materials and Methods

### Animals

All animal care and the experimental procedures were in accordance with the Guide for the Care and Use of Laboratory Animals published by the United States’ National Institutes of Health (NIH Publication No. 85-23, revised 1996). The Animal Care and Use Committee of Kaohsiung Medical University approved the protocol. Male Wistar rats (200–250 g) provided by the National Laboratory Animal Breeding and Research Center (Taipei, Taiwan) were housed under constant temperature and controlled illumination. Food and water were available ad libitum. In this experiment, we divided the rats into seven experimental groups: normal group (control), saline treated prevention group (Pv saline), liraglutide 75 μg·kg^−1^ (Novo Nordisk, Novo Alle, Bagsvaerd, Denmark) treated prevention group (Pv 75), liraglutide 200 μg·kg^−1^ treated prevention group (Pv 200), saline treated treatment group (Tx saline), liraglutide 75 μg·kg^−1^ treated treatment group (Tx 75), and liraglutide 200 μg·kg^−1^ treated treatment group (Tx 200).

### MCT induced PAH

All rats in the experimental groups except for the controls were given a single injection of MCT (60 μg·kg^−1^, i.p.) on day 0, which is known to be associated with the development of severe PAH within 3 weeks and a subsequent high mortality rate in rats. In the prevention groups, i.p. injections of saline (Pv saline), liraglutide 75 μg·kg^−1^ (Pv 75), and liraglutide 200 μg·kg^−1^ (Pv 200) were administered twice a day from day 1 to day 21 to reduce chronic PAH and the associated remodeling or hyperplasia. On day 21, the rats were anaesthetized for hemodynamic measurements. Lung tissues were then removed for Western blotting and immunocytochemistry, and heart tissues to evaluate right ventricular hyperplasia and hypertrophy. In the treatment groups, i.p. injections of saline (Tx saline), liraglutide 75 μg·kg^−1^ (Tx 75), and liraglutide 200 μg·kg^−1^ (Tx 200) were administered twice a day from day 22 to day 42 to treat chronic PAH and associated remodeling or hyperplasia. On day 42, the rats were anaesthetized for hemodynamic measurements. Lung tissues were then removed for Western blotting and immunocytochemistry, and heart tissues to evaluate right ventricular hyperplasia and hypertrophy.

### Hemodynamic measurements

Male Wistar rats were anesthetized with urethane (40 μg·kg^−1^, i.p.). Following tracheal cannulation, the anesthetized rats were mechanically ventilated, and the femoral artery was cannulated for continuous monitoring of mean arterial blood pressure and heart rate. Mean pulmonary artery pressure (mPAP) was measured in the PA of open chest rats. Hemodynamic parameters were recorded with a pressure transducer (ADInstruments Powerlab/4SP, ML 750, USA) connected to a pressure processor amplifier (Animal Bio Amp, ADInstruments, FE 136) and signal conditioner (ADInstruments, FE 221). Non fasting blood glucose measurements were obtained through a blood drop from a cannulated femoral artery using a handheld glucometer and One-Touch glucometer strips (FreeStyle Lite and FreeStyle Freedom Lite, Abbott, USA). After the blood had been collected from the femoral arteries into heparinized tubes, all experimental rats underwent saline perfusion followed by removal of the right middle lung which was stored at −80 °C until Western blot analysis. Perfusion with 10% formalin was then performed followed by removal of the right lower lung and heart which were soaked in 10% formalin and stored at 4 °C for future tissue sampling and assessment of the degree of right ventricular hypertrophy.

### Plasma concentration of ET-1

The plasma concentration of ET-1 was determined using an enzyme immunoassay kit according to the manufacturer’s instructions (Abcam, ab133030, USA). Blood was obtained from femoral arteries, followed by centrifugation at 3,000 rpm for 20 min at 4 °C to obtain plasma samples.

### Plasma concentration of cGMP

To measure the pulmonary release of cGMP, pulmonary arterial blood was collected in sample tubes coated with traces of heparin. The mixture was centrifuged at 3,000 rpm for 20 min, at 4 °C to obtain the plasma. The level of cGMP in pulmonary arterial plasma was determined by radioimmunoassay using a direct cGMP EIA kit according to the manufacturer’s instructions (ADI-900-014, Enzo Life Sciences, USA).

### Morphologic and immunocytochemistry analyses

Assessment of right ventricular hypertrophy (RVH) was made from the weight ratio of the right ventricular wall area to the left ventricular wall area plus the septum, the (RV/LV + S) ratio. The hearts of rats from each group were cut and soaked in formalin, dehydrated through graded alcohol, and embedded in paraffin wax. The heart tissue specimens fixed with formalin were embedded in paraffin, cut into 4-mm-thick sections and subjected to hematoxyline and eosin (H&E) staining prior to examination with a light microscope. Photomicrographs were obtained using a color digital camera mounted on a computer-interfaced light microscope (Nikon Eclipse, E600 microscopes, USA). The relative weight ratio was calculated as RV/left ventricle + septum (RV/LV + S) using H&E staining. Measurements were obtained using HistoLab Image-Pro Plus software (Media Cybernetics, USA).

To confirm vascular hyperplasia in the PA rings, right lung tissue sections from the rats were prepared as described above and stained with H&E. Microphotographic measurements of the PA in the lung sections were performed using Nikon Eclipse E600 microscopes interfaced with Image-Pro Plus software. Medial thickness (micrometers) and medial wall area (calculated as the area between the internal elastic lamina and the adventitia in micrometers) of the muscular layer of the pulmonary arteries were determined.

The immunocytochemical analysis of increased levels of eNOS induced by liraglutide was performed as follows. The eNOS antibody (rabbit anti-eNOS polyclonal antibody, Abcam, 5589, USA) (1:100) was selected for immunostaining of the right lung sections. The lungs were perfused with saline followed by 10% formalin and then placed in 10% formalin for paraffin embedding. The lung tissues were cut into 3–5 mm sections on slides, de-waxed in 100% xylene, and then rehydrated in graded alcohol solutions. Throughout the procedure, the slides were washed as appropriate in phosphate buffered saline (PBS). Antigen retrieval was performed by microwave treatment for eNOS immunostaining. The sections were treated with 3% H_2_O_2_ (10 min) to inhibit endogenous peroxidase and incubated with normal serum (60 min) to reduce non-specific binding of secondary antibodies. The sections were then incubated at 4 °C overnight with eNOS antibodies. After washing off unbound primary antibodies, the sections were incubated with secondary biotinylated antibodies against mouse or rabbit (DAKO, K5007, USA) for 60 min, followed by incubation in avidin/biotin/horseradish peroxidase complex (DAKO) for 30 min. Subsequently, images were collected by confocal laser scanning microscope (Olympus Fluoview FV1000, Olympus Optical Co, Tokyo, Japan).

### Preparation of PASMCs

PASMCs were isolated from the first branch of the pulmonary artery of 8-week-old male Wistar rats. The rats were euthanized under deep anesthesia before their heart and lungs were removed and rinsed several times in PBS. The pulmonary arteries were then segregated in a sterile manner. The outer sphere was peeled off and the microtubule was snipped, while the endothecia was shaved lightly two-to-three times to remove the endothelial cells. The tunica media was prepared into scraps in DMEM.

The cells were cultured in DMEM supplemented by 10% FBS, 2 mM glutamine, 100 U·mL^−1^ penicillin, and 100 mg·mL^−1^ streptomycin in a Petri dish (Corning 430167, diameter 100 mm, Corning, Inc., Tewksbury, MA, USA) and incubated at 37 °C (5% CO_2_, 95% air). The culture medium was changed every 3 days and the cells were sub-cultured until confluence. The primary cultures of two-to-four passages were used in the experiments. The purity of PASMC was confirmed by immunofluorescence staining for α-smooth muscle actin (>95% of cells stained positive) and lack of staining for vimentin under confocal microscope. Before each experiment, serum starvation was achieved by 1% FBS for 48 h to synchronize the cells into quiescence.

### Determination of cell migration

Two migration assays were performed as previously described^30^. First, the PDGF-BB-mediated PASMCs migration assay was performed using a Boyden chamber. Briefly, PASMCs (2 × 10^4^ cells) were loaded into the upper compartment. Meanwhile, in the lower compartment, PDGF-BB (20 ng·mL^−1^) was added to the dissolved in serum-free DMEM with various concentrations of GLP-1 receptor agonist liraglutide (1–10 nM, pre-treatment for 1 h). After 24 h, the non-migrated cells on the upper membrane surface were removed and those on the lower surface were fixed in methanol and stained with Giemsa. The number of cells per six high-power fields (200 × HPF) was counted and the mean number was used for migration activity.

For the wounding assay, PASMCs that had been grown to confluence in 6-well cell culture plates. FBS was removed from the media and was replaced by serum-free media. A plastic pipette tip was drawn across the center of the plate to produce a clean wound area 24 h after serum depletion. Multiple photographs of the wound were obtained 24 h post-wounding in serum-free medium (control) or in the presence of 20 ng·mL^−1^ PDGF-BB. The effect of liraglutide was analyzed by adding it to the culture medium 1 h before adding PDGF-BB. The migration distance between the leading edge of the migrating cells and the edge of the wound was measured for comparison.

### Assessment of cell cycle by flow cytometry

PASMCs were synchronized at the G_0_-phase by serum starvation for 48 h. After replenishing with fresh DMEM supplemented by 1% FBS, the cells were pre-treated with various concentrations of liraglutide (1–10 nM) for 10 min. PDGF-BB (20 ng·mL^−1^) was then added to allow the progression of the cell cycle for 48 h. The cells were trypsinized, centrifuged at 1,000 rpm for 5 min, washed twice with cold PBS, and treated with RNase A (10 mg·mL^−1^). The DNA was stained with propidium iodide (50 mg·mL^−1^) for 30 min at 37 °C and 5 × 10^3^ cells were analyzed by flow cytometry.

### Protein expression by Western blotting analysis

Whole right lung tissues of the rats were isolated and cut into small chips to extract the protein. To measure the protein expression levels, total proteins were extracted, and Western blotting analysis was performed as described previously^30^,^31^. Mouse anti-eNOS monoclonal antibody 1:1000 (Transduction, N 30020, USA), rabbit anti-sGC polyclonal antiserum1:1000 (Cayman, 160890, USA), rabbit anti-PKG monoclonal antibody1:1000 (Calbiochem, 370652, USA), mouse anti-Rho kinase monoclonal antibody1:1000 (Upstate, 05–778, USA), and the loading control protein rabbit anti-GAPDH polyclonal antibody 1:1000 (Sigma, A5441, USA) and anti-β-actin polyclonal antibody 1:1000 (Sigma, A2066) were used. The expression levels of the proteins were analyzed via chemiluminescence and quantified using ImageJ Software.

### Statistical analysis

The results were expressed as mean ± standard error of the mean (SEM). Data were analyzed in SigmaPlot software (version 8.0, SPSS Scientific, Chicago, IL, USA) by Student’s t-tests or one-way ANOVA, and Student-Newman-Keuls method was used for comparisons of group means. A p-value less than 0.05 was considered to be statistically significant.

## Additional Information

**How to cite this article**: Lee, M.-Y. *et al*. Liraglutide prevents and reverses monocrotaline-induced pulmonary arterial hypertension by suppressing ET-1 and enhancing eNOS/sGC/PKG pathways. *Sci. Rep.* **6**, 31788; doi: 10.1038/srep31788 (2016).

## Supplementary Material

Supplementary Information
