# Supplementary material for: Liraglutide prevents and reverses monocrotaline-induced pulmonary arterial hypertension by suppressing ET-1 and enhancing eNOS/sGC/PKG pathways
Source: Sci Rep. 2016 Sep 1;6:31788. doi: 10.1038/srep31788 (PMC5007506; doi:10.1038/srep31788)

Supplementary information

Liraglutide prevents and reverses monocrotaline-induced pulmonary arterial hypertension by suppressing ET-1 and enhancing eNOS/sGC/PKG pathways

Mei-Yueh Lee[1,2,3], Kun-Bow Tsai[4], Jong-Hau Hsu[5,6], Shyi-Jang Shin[2], Jiunn-Ren Wu[5,6*] & Jwu-Lai Yeh[1,7,*]

The following graphics are the original Western blot of figures 4 and 7.

Figure 4A eNOS

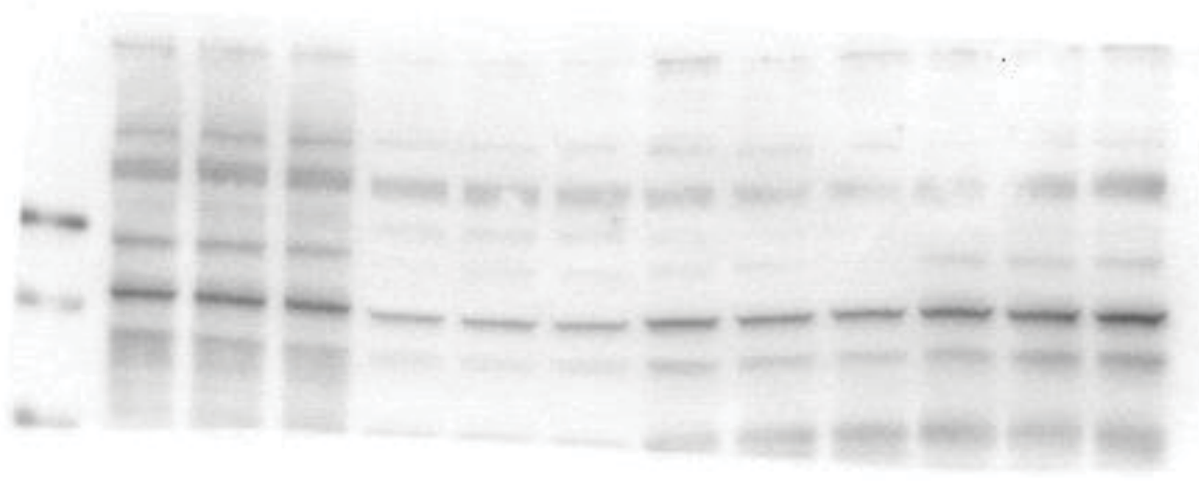

Figure 4A GAPDH

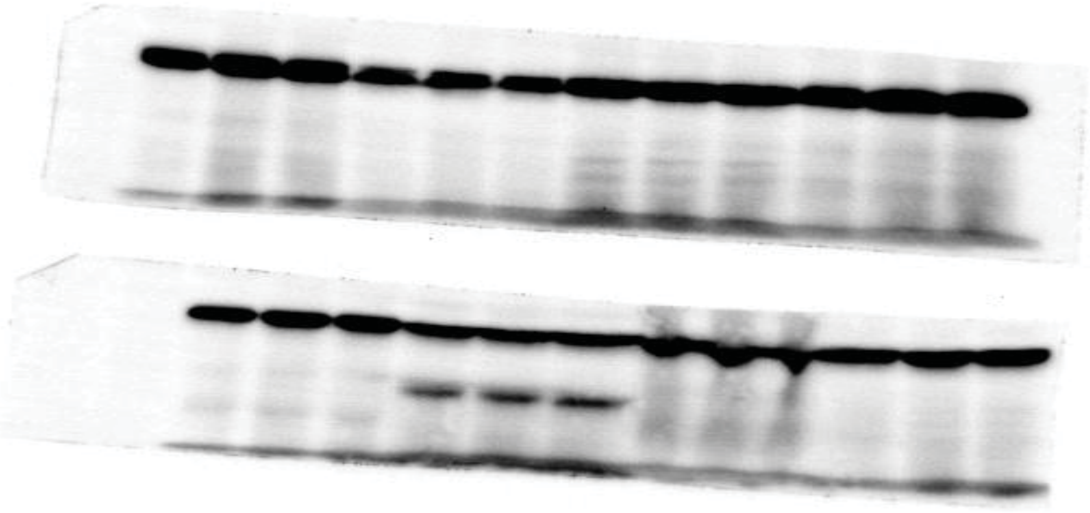

Figure 4A PKG

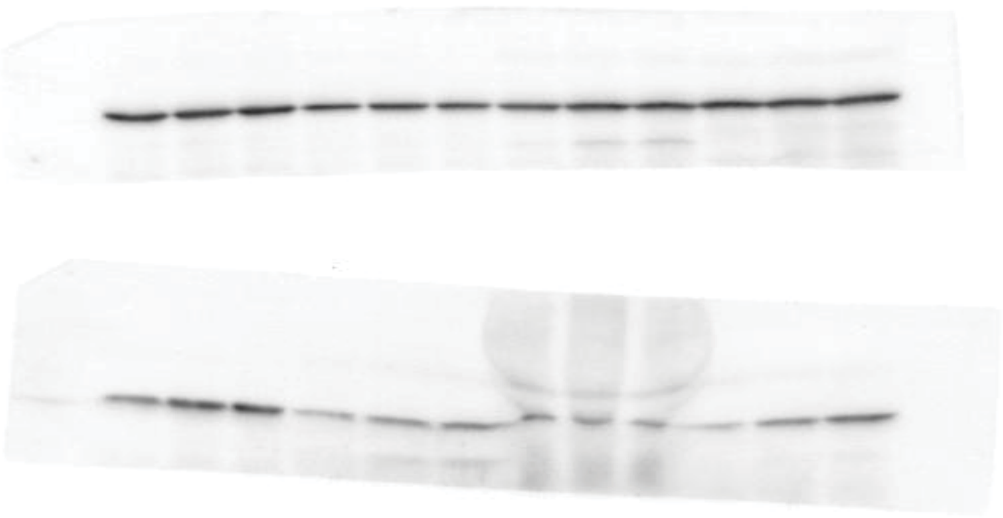

Figure 4A ROCK

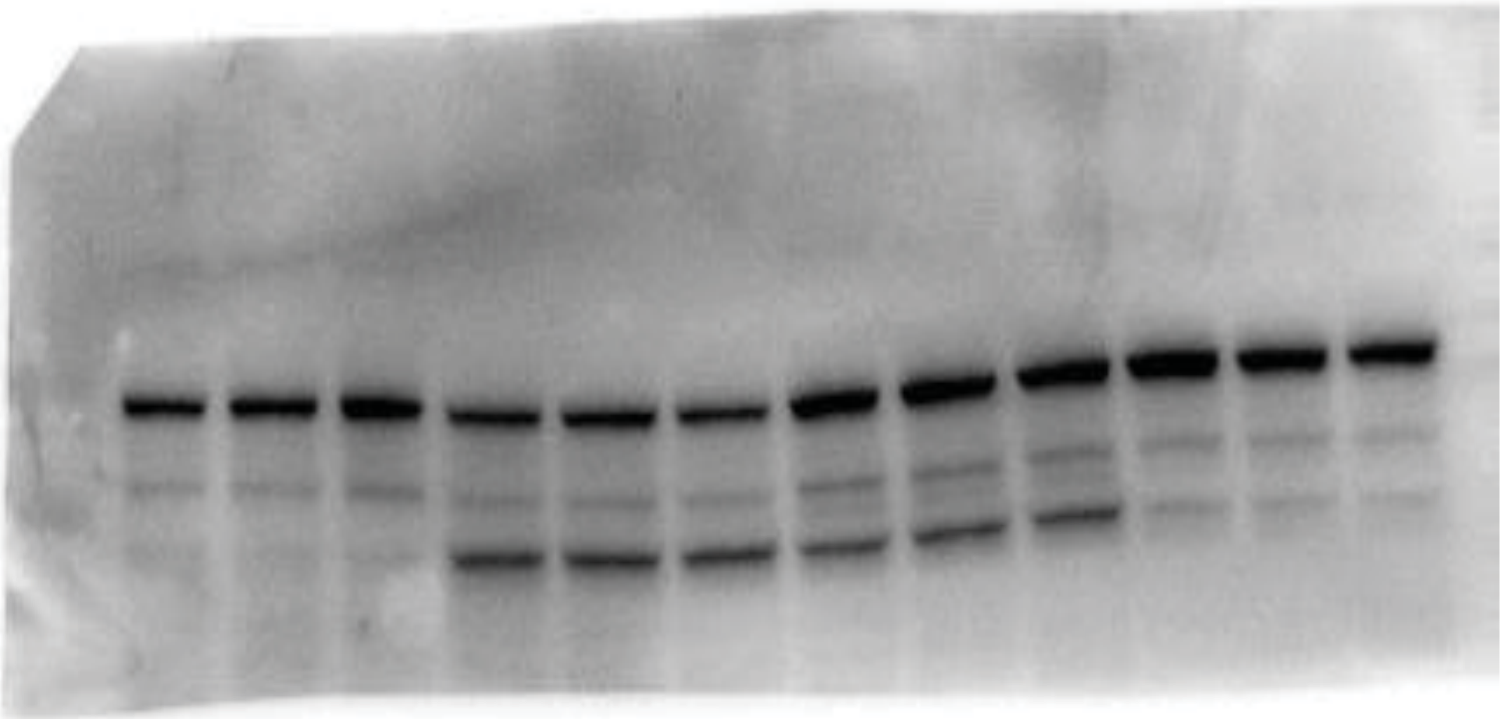

Figure 4A SGC

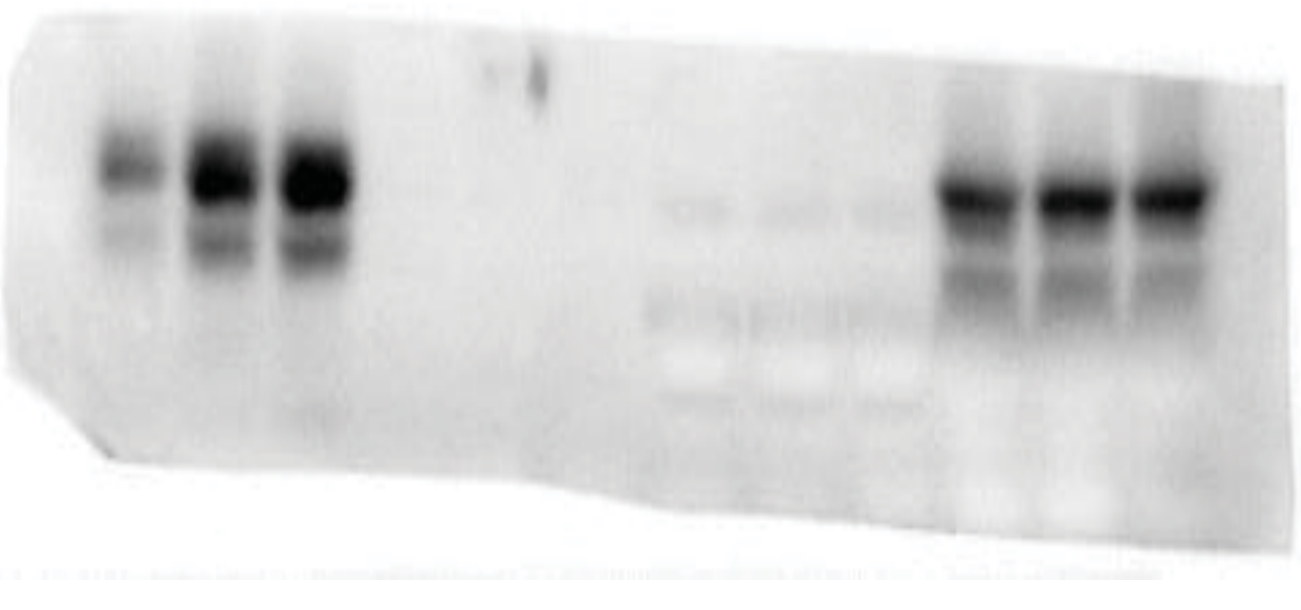

Figure 4B eNOS

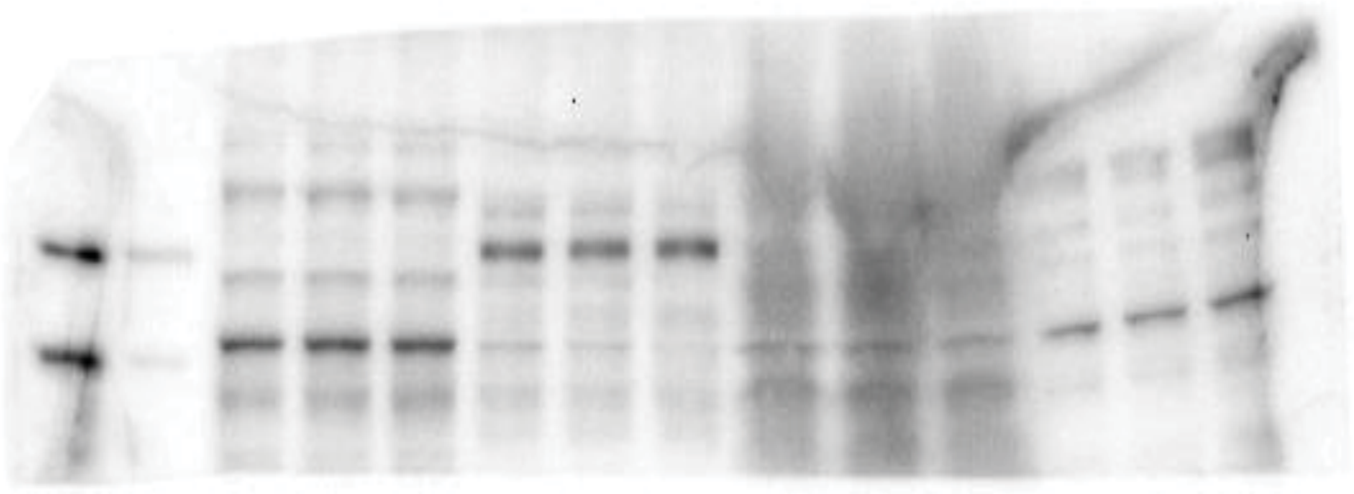

Figure 4B GAPDH

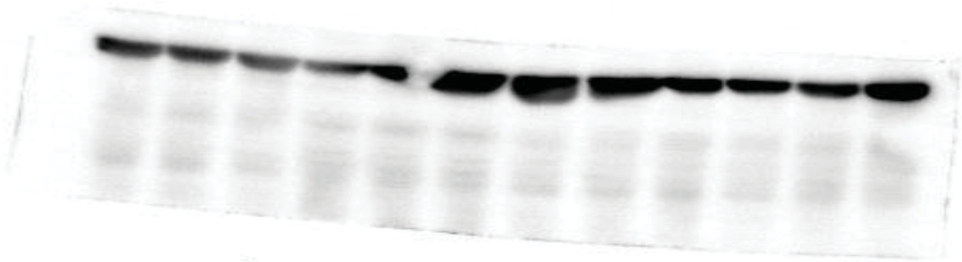

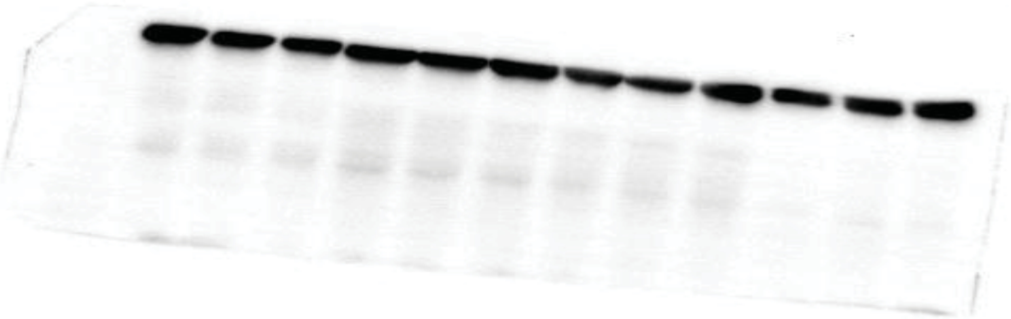

Figure 4B PKG

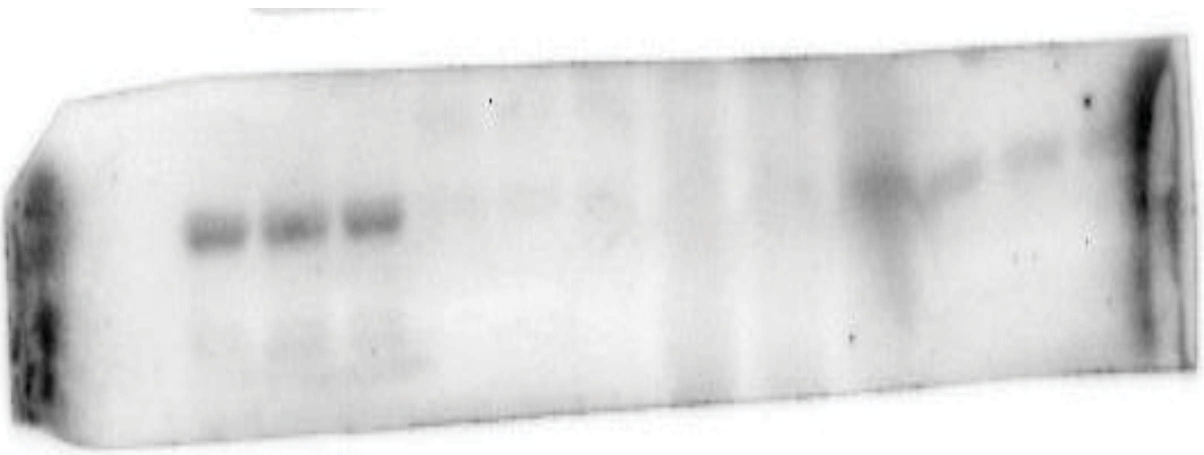

Figure 4B ROCK

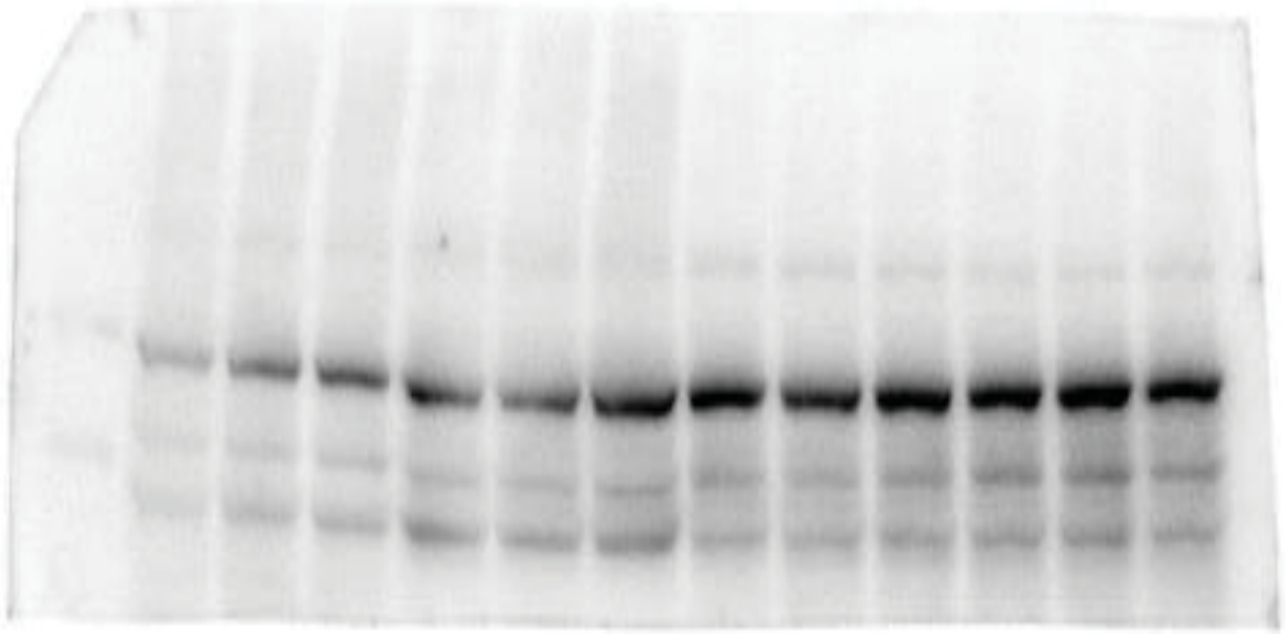

Figure 4B SGC

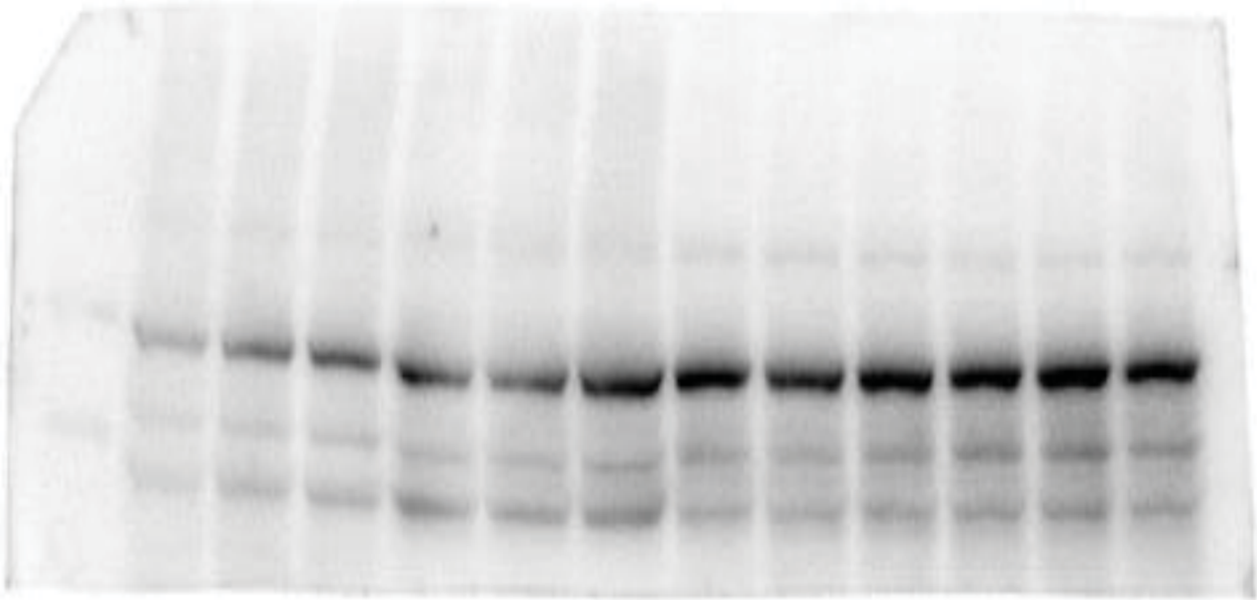

Figure 7 β actin

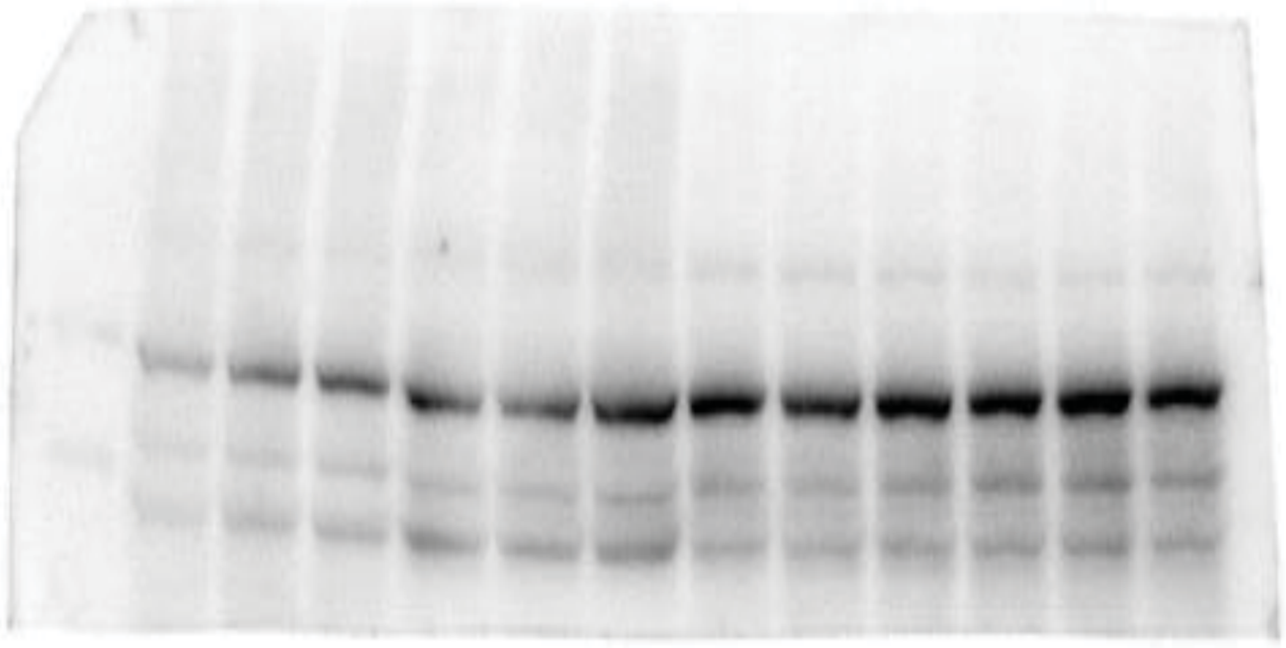

Figure 7 eNOS

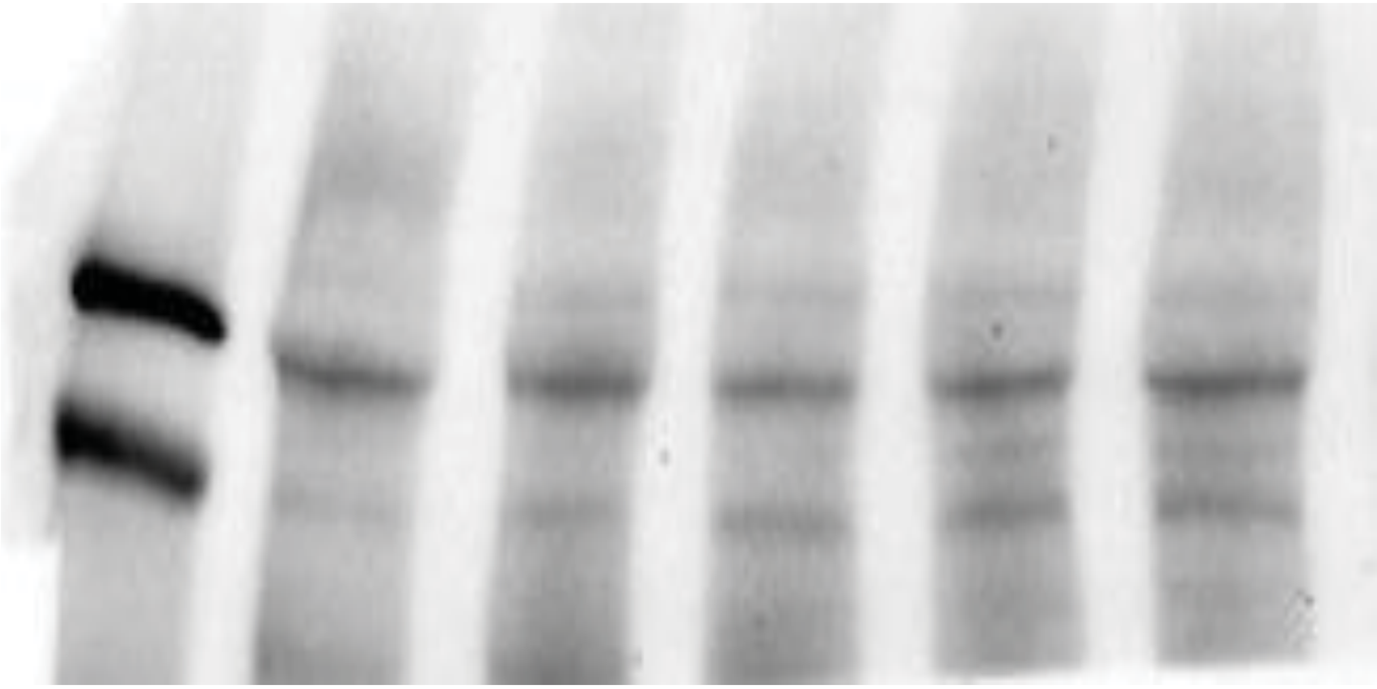

Figure 7 PKG

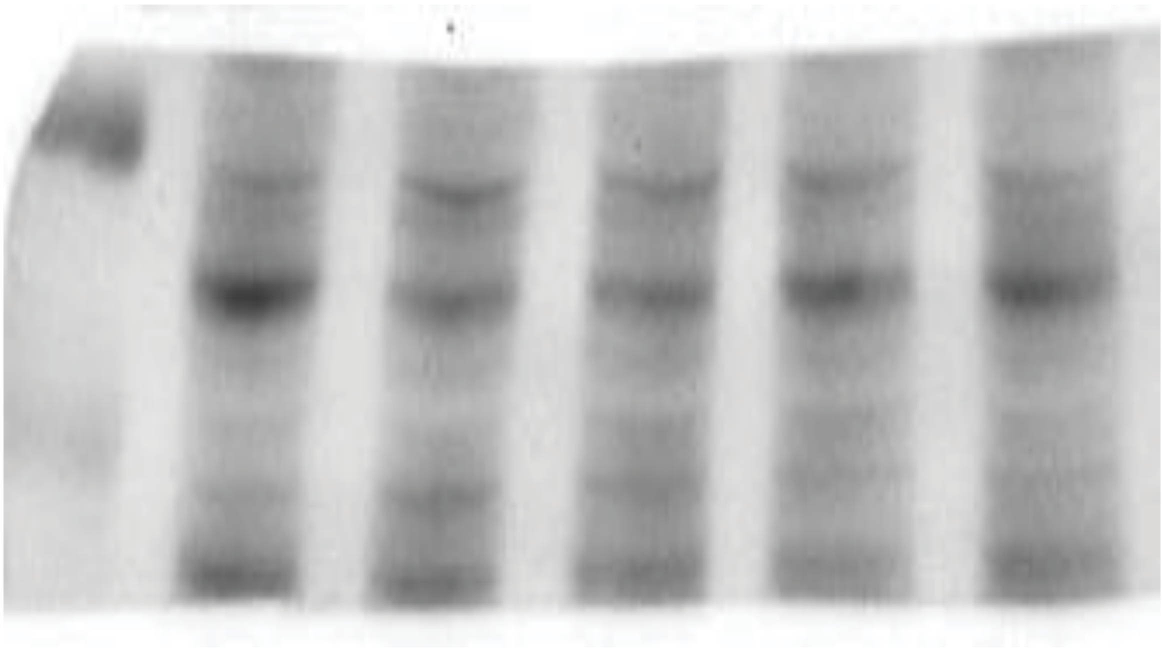

Figure 7 ROCK

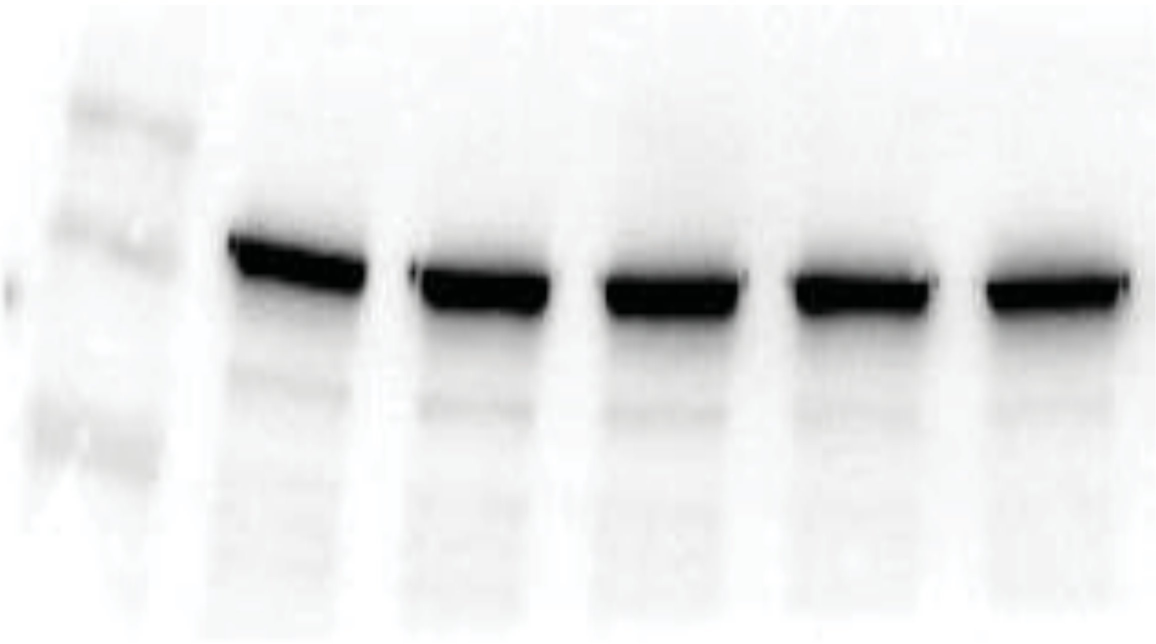

Figure 7 SGC

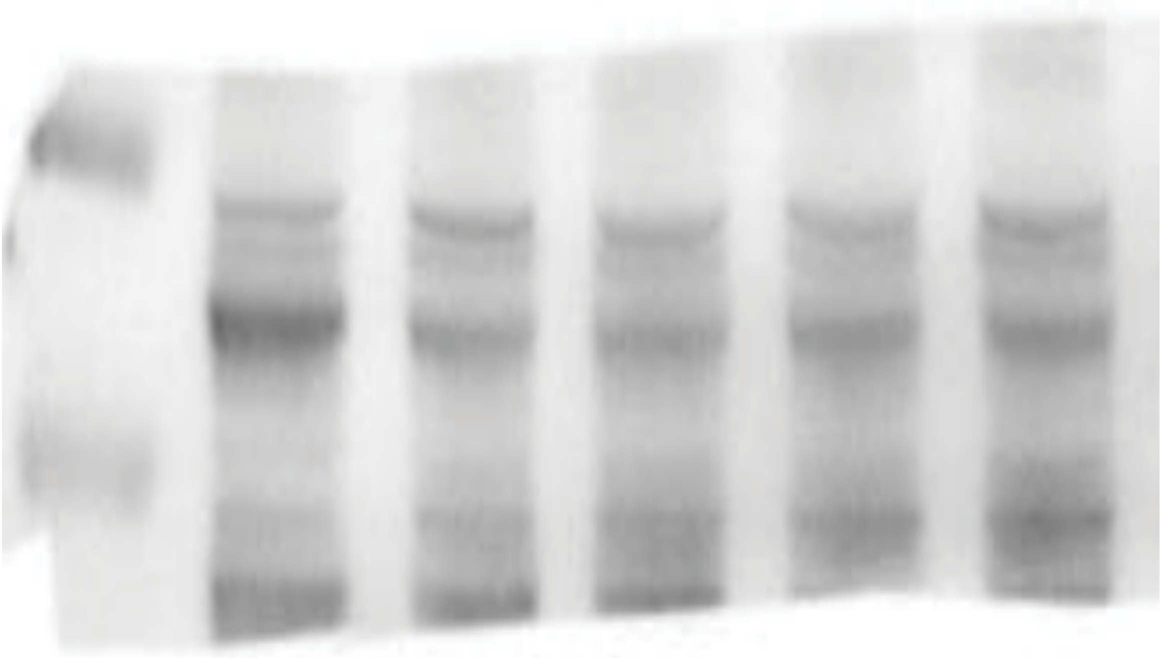

Supplement: Supplementary Information [file srep31788-s1.pdf]
